# Transcriptomic analysis of eggs, rediae and cercariae reveal stage-specific adaptations in the rumen fluke *Calicophoron daubneyi*

**DOI:** 10.1186/s12864-026-13271-z

**Published:** 2026-09-02

**Authors:** Leah R. Knöpfle, Raúl O. Cosentino, Markus R. Schmidt, Verena K. Elbert, Sandra Haug, Frank Weber, Antonio Vazquez Perera, T. Nicolai Siegel, Markus Meissner

**Affiliations:** 1 Clinic for ruminants with Ambulatory and Herd Health Services, Ludwig-Maximilians-Universität München, Oberschleißheim, Germany; 2 Chair for Experimental Parasitology, Faculty of Veterinary Medicine, Ludwig-Maximilians-Universität München, Munich, Germany; 3 Biomedical Center, Division of Physiological Chemistry, Faculty of Medicine, Ludwig-Maximilians-Universität München, Munich, Germany; 4 Institut de Recherche pour le Développement, Université de Montpellier, Montpellier, France

**Keywords:** *Calicophoron daubneyi*, Rumen fluke, Transcriptome, Rna-sequencing, Stage-specific gene expression, Different developmental stages

## Abstract

Rumen flukes, particularly the trematode *Calicophoron daubneyi*, are emerging parasites of livestock in Europe, yet transcriptomic insights into their environmental and intermediate host stages remain limited. Here, we present a comprehensive transcriptomic analysis of eggs at three distinct developmental stages (freshly excreted, early developmental and eye-spot stages), as well as rediae and cercariae, of *C. daubneyi*. High-quality RNA-sequencing (RNA-seq) datasets revealed both shared and stage-specific transcriptional profiles with each developmental stage exhibiting its own distinct expression pattern. Subsequent GO-Term enrichment analyses revealed that fully embryonated eggs in eye-spot-stage especially upregulated genes related to cilia assembly, movement and motility, reflecting preparation for miracidial hatching and host-seeking behavior. Rediae showed enhanced transcription of genes involved in diverse metabolic and biosynthetic processes, supporting rapid asexual proliferation within the snail intermediate host. Cercariae exhibited predominant upregulation of genes associated with signal transduction and energy metabolism, indicating the adaptation to its changing environmental conditions. These findings provide the first transcriptomic insights into the biology of *C. daubneyi* outside the definitive host, reveal molecular mechanisms underlying development, transmission and adaptation to a changing environment and identify stage-specific genes as potential targets for interventions aimed at disrupting the parasites life cycle and controlling rumen flukes in the future.

**Supplementary Information:**

The online version contains supplementary material available at https://doi.org/10.1186/s12864-026-13271-z.

## Background

Paramphistomes, commonly known as rumen flukes, are of concern to livestock producers worldwide due to their potential to cause disease and mortality in cattle, sheep and goats. Once confined to the subtropics and tropics, rumen flukes have been spreading to more temperate climate zones in recent decades [1, 2]. Numerous European countries documented a substantial rise in both the prevalence and severity of rumen fluke infections [3–6]. As a result, paramphistomidosis is now regarded as an emerging parasitic disease in Europe [2]. Species identification revealed *C. daubneyi* as the predominant species throughout Europe and Germany, which shares the same intermediate host, the mud snail *Galba truncatula*, with the well-established common liver fluke *Fasciola hepatica* [7–12].

Classified as a digenean trematode, *C. daubneyi* undergoes a complex lifecycle requiring an intermediate molluscan host to complete its maturation. Unembryonated parasitic eggs, passed in feces, develop in a preferably warm, moist and bright environment within few weeks [13, 14]. Subsequently, hatched ciliated miracidia penetrate the snail and undergo a series of asexual reproductive stages, transforming into sporocysts, rediae, and procercariae. Finally, free-swimming cercariae emerge from the snail to encyst as metacercariae on vegetation, which are then ingested by the mammalian host [15, 16].

The clinical picture of paramphistomidosis is mainly caused by a severe intestinal infection with juvenile parasitic stages that adhere to the mucosa of the small intestine [13, 17]. Diarrhea, anorexia, dehydration, weakness and even death have been described as possible consequences [18–22]. On the other hand, the chronic ruminal phase, caused by persisting adult flukes in the rumen, usually remains clinically unremarkable, although pathological changes can still be observed [4, 13, 23]. Since treatment options are very limited, as oxyclozanide is the only anthelmintic considered effective but is not approved for this indication, prevention of infection will play a crucial role in the control of paramphistomidosis in the future [24, 25].

‘Omic’ datasets have already greatly advanced studies on other trematodes, allowing for comparative analysis between different life-cycle stages and species. During the study of several snail intermediate host stages of trematode *Fasciola gigantica*, for example, a clear correlation was established between stage-specific gene expression patterns and the associated changes in external circumstances such as finding an intermediate host or survival in the environment [26]. Furthermore, in *F. hepatica*, differences in gene transcription could be recognized in the development of eggs [27]. On the other hand, especially compared to other flukes, our basic biological knowledge about *C. daubneyi* is very limited. Previous transcriptome analyses of *C. daubneyi* have provided an initial basis to identify stage-specific gene expression, but have exclusively focused on the adult fluke stages and those found in the mammalian definitive host [28, 29]. However, in order to prevent the spread of paramphistomidosis in the long term, a deeper understanding of the parasite’s survival strategies in the environment and its interaction with the intermediate snail host is essential.

In this study, we present the first transcriptome of intermediate host stages and stages in the environment of *C. daubneyi* including eggs of three different developmental stages, rediae and cercariae. Our data suggest that the parasite adapts to the changing conditions of its complex developmental cycle through distinct transcriptional changes, and that some developmental stages face greater adaptive challenges than others. These findings not only highlight the importance of molecular adaptation strategies of parasitic trematodes, but can help to introduce parasite control measures to prevent transmission via the intermediate host and enhance our understanding of epidemiological factors affecting life-cycle stages outside the mammalian host.

## Methods

### Collecting of parasite material

A total of approximately 1,200 adult rumen flukes were collected alive using anatomical forceps from the rumen of a naturally infected Galloway bull, which was examined at a local abattoir (Schwabmünchen, Germany). The parasites were washed in sterile 0,9% physiological saline solution (B. Braun, Melsungen) at 37 °C and transported in a preheated collection container to the laboratory, ensuring temperature stability.

In the laboratory, the live flukes were incubated in fresh NaCl solution at 38 °C. The solution was renewed every one to two hours and the vitality and motility of the flukes were visually monitored. Dead individuals were preserved. The collected NaCl supernatant contained the excreted eggs, which were washed and concentrated by repeated centrifugation. In total, approximately 750,000 eggs were obtained.

The harvested eggs were divided into three groups, each consisting of at least 10,000 eggs, with three replicates. Eggs collected immediately after shedding (T0) were used for RNA extraction on the same day. Eggs from groups T7 (developing eggs) and T14 (eye-spot-stage) were incubated in distilled water without light at 27 °C for 7 and 14 days [30]. After each incubation period, egg development was examined microscopically; dead or poorly developed eggs were discarded and RNA was extracted on the same day.

To obtain miracidia for snail infections, fecal samples from infected cattle were examined by sedimentation and eggs were identified via coproscopy and collected. The eggs were incubated at 27 °C for 12 to 15 days and miracidia were induced to hatch by light stimulation. As previously described in Elbert et al. (2024) *Galba truncatula* snails, bred under controlled conditions in the laboratory, were infected with 5 miracidia each and dissected starting from day 21 post-infection (p.i.) for the collection of rediae, and from day 55 p. i. for the collection of cercariae. The infection trials were conducted in collaboration with the University of Perpignan (Perpignan, France). Three replicates, each containing 50 rediae or cercariae, were washed and stored in lysis buffer at -80 °C until final RNA extraction.

### Species confirmation

To identify the species *C. daubneyi* by PCR, the DNA of the eggs, infected snails and adult flukes was extracted using the DNeasy Blood & Tissue Kit (QIAGEN). The primer pair Cd-Cox Neu (forward: GTTTGTGTGGGTTTGCCACGG; reverse: CTACCCCAAGCAGCCACTAC, 169 bp) was used for the PCR (Jones et al., 2015). A previously verified *C. daubneyi* DNA served as positive control and nuclease-free water as negative control. PCR was performed using the Professional Thermocycler (Biometra Analytik Jena AG) and qPCRsoft 3.4 software. DNA analysis was performed using the 4150 TapeStation System and D1000 ScreenTape (Agilent Technologies). Since none of the samples analysed deviated from the positive control by a maximum of +/- 10 bp, the analysis confirmed the species *C. daubneyi* for all analysed samples.

### RNA extraction, library preparation and sequencing

For total RNA extraction, the lysis buffer included in the RNA extraction kit (RNA Mini Kit, Bio-&SELL, Nuremberg, Germany) was added to each sample before being mechanically homogenized (Lysis Tubes P with 2.4–2.8 mm Ceramic beads, Homogenizer SpeedMill PLUS, Analytic Jena, Switzerland) and further processed according to the manufacturer’s instructions. In addition, a DNAse inactivation step was performed using the DNAse I column digestion kit (Bio&SELL, Nuremberg, Germany).

Subsequent steps, library preparation and sequencing, were carried out by IMGM Laboratories (Medicover Integrated Clinical Services, Martinsried, Germany). Quantity and integrity of the RNA samples were measured with the 2100 Bioanalyzer (Agilent Technologies). RNA extracted from the examined developmental stages was of high quality, with mean RIN values of 9.1. Library preparation was performed on 15 ng of total RNA in triplicate samples (Eggs T0, Eggs T14, Rediae, Cercariae). First, a poly(A) RNA selection step was carried out using the NEBNext^®^ Poly(A) mRNA Magnetic Isolation Module (New England Biolabs). From the enriched material, RNA-Seq libraries were prepared using the NEBNext^®^ Ultra™ II Directional RNA Library Prep Kit (New England Biolabs) according to the manufacturer’s instructions with fragments amplified during a 15-cycle PCR reaction. All libraries were quality controlled using the 2100 Bioanalyzer (Agilent Technologies) and quantified using Qubit^®^ ds DNA HS Assay Kit (Thermo Fisher Scientific). Finally, libraries were pooled and sequencing was performed according to the standard workflow on an Illumina NovaSeq^®^ 6000 next generation high-throughput sequencing platform (single-end sequencing, 75 bp-long reads) using the sequencing-by-synthesis (SBS) technology. Sequencing produced a total of 523,227,236 raw reads, which after trimming resulted in 518,479,102 clean reads.

### Data processing and quality filtering

Image processing was performed on the NovaSeq^®^ 6000 instrument using the Real Time Analysis Software (RTA, v.3.4.4). The Illumina Sequence Analysis Viewer (SAV, v.2.4.7) was used for imaging and evaluation of the sequencing run performance. Primary analysis of the raw Illumina data including signal processing to fastq files and de-multiplexing of indexed reads was performed with the bcl2fastq software tool. The fastq files were quality controlled using the CLC Genomics Workbench QC report tool (Qiagen, v.23.0.4). Adaptor sequences, low quality sequences (quality limit = 0.05) and ambiguous nucleotides (maximum number of ambiguities = 2) were removed.

### De novo transcriptome assembly

RNA-seq data generated from the multiple life-cycle stages of *C. daubneyi* were pooled to construct a comprehensive reference transcriptome. De novo transcriptome assembly was performed using Trinity (v.2.15.1) with default parameters [31]. The resulting transcriptome was subsequently evaluated for completeness and quality using multiple complementary approaches. To assess read representation in the assembly, RNA-seq reads from each sample were independently aligned back to the assembled transcripts using Bowtie2 (v.2.4.5), and overall alignment rates were calculated [32]. Assembly completeness was further evaluated using BUSCO (v.5.8.2) in transcriptome mode (euk_tran), using the eukaryota_odb10 lineage dataset to assess the presence of conserved single-copy orthologs expected for eukaryotes [33]. Assembly contiguity and expression support were assessed using the ExN50 metric, which relates transcript length distribution to expression level. Expression-weighted contiguity (ExN50) statistics were calculated at the gene level, based on the longest isoform per Trinity gene. The Ex95 N50 value, representing the N50 of the most highly expressed 95% of assembled genes, was used as an indicator of contiguity for the biologically relevant core transcriptome.

Open reading frames (ORFs) were predicted from the assembled transcripts using TransDecoder (v.5.7.1) [31], and predicted protein sequences were retained for downstream functional annotation. Functional annotation of the transcriptome was performed using Trinotate (v.4.0.2), integrating results from BLASTX and BLASTP searches (blast v.2.16.0) against the Swiss-Prot database, as well as protein domain identification using HMMER (v.3.1b2) hmmscan against the Pfam database [34–38]. Annotation results were compiled into a unified Trinotate report, providing functional assignments at both the transcript and protein levels.

### Transcript expression quantification and clustering

Transcript abundance was quantified for each RNA-seq library using Salmon (v.1.6.0), as implemented within the Trinity pipeline, using the de novo assembled transcriptome as reference [39]. Transcript-level abundance estimates were summarized to the gene level using Trinity utility scripts to enable downstream gene-centric analyses. Patterns of gene expression across life-cycle stages were initially explored at the gene level by visualizing expression intersections using UpSetPlot (v.0.9.0) [40]. Differential expression analysis was subsequently performed at the gene level using DESeq2 (bioconductor-deseq v.1.34.0), following the Trinity differential expression workflow [41]. Pairwise comparisons were conducted between life-cycle stages, and genes were considered differentially expressed if they met the thresholds of a false discovery rate (FDR) < 0.001 and an absolute log2 fold change > 2.

For gene clustering analysis, differentially expressed genes from all pairwise comparisons were pooled, retaining up to a maximum of 1,000 genes per comparison to avoid overrepresentation of highly contrasted comparisons. Hierarchical clustering of gene expression profiles was performed within the Trinity framework using normalized expression values across all samples. Expression clusters were defined by cutting the hierarchical clustering tree at 30% of its height (--Ptree 30). This threshold was empirically selected to balance cluster resolution and interpretability, resulting in a total of 16 expression subclusters with distinct life-stage expression patterns.

### Gene ontology enrichment analysis

Gene ontology (GO) enrichment analysis was conducted on individual expression subclusters to identify biological processes associated with stage-specific or shared transcriptional programs. GO annotations were based on the GO database release 2024-03-28. GO enrichment was performed using GOseq (v.1.46.0) within the Trinity/Trinotate framework, which accounts for transcript length bias inherent to RNA-seq data [42]. The background gene set used was the complete set of differentially expressed genes across all subclusters (3,736 genes). The statistical test used was the Wallenius approximation, which accounts for transcript length bias when testing for GO term enrichment. The resulting p-values were adjusted for multiple testing using the Benjamini-Hochberg false discovery rate (FDR). For each subcluster, over-represented GO categories were identified relative to the background set of expressed genes. Enrichment analyses were restricted to the Biological Process ontology. GO terms were considered significantly enriched if they met the following criteria: FDR ≤ 0.05, a minimum of three differentially expressed genes assigned to the category, and a total category size of fewer than 500 genes. Broad, non-informative GO terms (e.g., “biological process”, “cellular process”, and “metabolic process”) were excluded from downstream visualization and interpretation. GO enrichment results were visualized using dot plots, in which the gene ratio (number of differentially expressed genes in a GO term relative to the total number of genes annotated to that term) was plotted against GO categories. Dot size reflected the number of genes associated with each GO term, and color encoded the adjusted p-value (FDR).

## Results

**Fig. 1:**
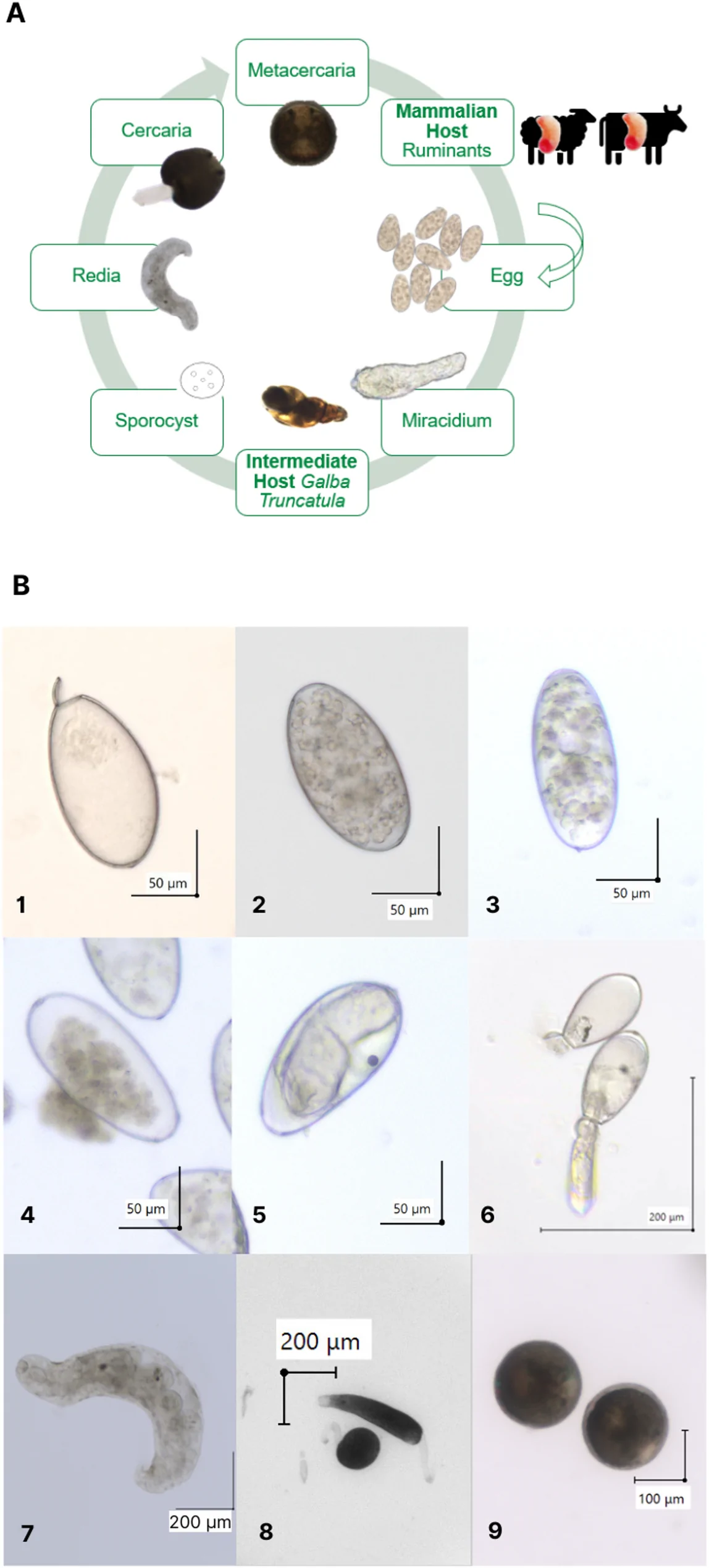
**A** Graphical representation of the *C. daubneyi* lifecycle with focus on the environmental and intermediate host stages. **B** Microscopic images of different developmental stages of *C. daubneyi.* 1: Empty eggshell after miracidium hatching. 2: Freshly excreted egg from an adult rumen fluke. 3: Egg at the onset of development with initial cell organization. 4: Dead egg with released contents. 5: Fully embryonated egg in the eye-spot-stage containing a miracidium ready to hatch. 6: Empty eggshell and miriacidium during hatching process. 7: Redia. 8: Fully developed cercariae. 9: Encysted metacercariae

The life cycle of *C. daubneyi* in both the environment and the intermediate host comprises unembryonated eggs excreted in feces, which under optimal environmental conditions undergo cell division to develop into miracidia, sporocysts, rediae, cercariae, and finally, metacercariae (Fig. 1A). To verify developmental progression and ensure accurate stage assignment for transcriptomic analysis, eggs at three developmental stages, as well as rediae and cercariae, were examined microscopically to characterize these processes.

Eggs collected at day 0 (freshly excreted from adult flukes) were homogeneous in size, shape, and appearance and were unfertilized. By Day 7, early cell organization was evident within the eggs. Approximately 63% of day 14 eggs had reached the embryonated stage, with fully formed, motile miracidia recognizable within the eggs, including the eye spot stage. Subsequent light exposure induced hatching in 80% of the fully developed miracidia.

Rediae and cercariae were obtained by dissecting infected snails. All intermediate stages were observed concurrently within the snail body and were distinguishable under microscopy, demonstrating the sequential developmental progression of *C. daubneyi* (Fig. 1B).

### De novo transcriptome assembly of *C. daubneyi*

To generate a comprehensive reference transcriptome spanning multiple developmental stages, a *de novo* transcriptome assembly of *C. daubneyi* was generated from the pooled RNA-seq data of the examined developmental stages using the Trinity software [43]. The final assembly comprised 172,970 transcript isoforms corresponding to 114,812 genes, with a GC content of 45.4% (Table 1). The median and average transcript lengths were 335 bp and 696 bp, respectively, with an overall N50 of 1,286 bp calculated across all transcript contigs. When considering only the longest isoform per gene, the assembly retained an N50 of 666 bp, reflecting the inclusion of multiple alternatively spliced isoforms in the full transcript set.

**Table 1:** Summary statistics of the *C. daubneyi* de novo transcriptome assembly and annotation. Summary statistics describing the de novo transcriptome assembly of *C. daubneyi* generated using Trinity from combined RNA-seq data across multiple life stages

Metric	Value
Assembly statistics (all transcripts)	
Total genes	114,812
Total transcripts	172,970
Total assembled bases (Mb)	120.4
N50 (bp)	1,286
Mean transcript length (bp)	696
Median transcript length (bp)	335
GC content (%)	45.4
Assembly statistics (longest isoform per gene)	
Total transcripts	114,812
Total assembled bases (Mb)	59.4
N50 (bp)	666
Ex95 N50 (bp)	1,570
Genes contributing to Ex95	16,679
Completeness (BUSCO eukaryota_odb10)	
Complete BUSCOs (%)	93.7
Single-copy BUSCOs (%)	25.9
Duplicated BUSCOs (%)	67.8
Fragmented BUSCOs (%)	3.1
Missing BUSCOs (%)	3.2
Read support	
Mean read mapping rate (%)	~ 93
Read mapping rate range (%)	84–97
Coding potential (TransDecoder)	
Predicted proteins	48,512
Complete ORFs (%)	42.3
Functional annotation (Trinotate)	
Annotated transcripts (%)	21.3
Transcripts with Swiss-Prot BLASTX hit (%)	18.4
Proteins with Swiss-Prot BLASTP hit (%)	56.2
Proteins with Pfam domain (%)	60.1

To evaluate the completeness of the transcriptome assembly, the presence of evolutionarily conserved genes was assessed using the tool Benchmarking Universal Single-Copy Orthologs (BUSCO [DOI: https://doi.org/10.1007/978-1-4939-9173-0_14]) and comparing to the eukaryotic-specific gene dataset (eukaryota_odb10). BUSCO identified 93.7% of expected conserved genes as complete, including a high proportion of duplicated BUSCOs (67.8%), consistent with transcript redundancy and alternative isoform representation inherent to de novo transcriptome assemblies. Additionally, read support for the assembly was high, with 84–97% of RNA-seq reads from individual samples successfully mapping back to the assembled transcriptome, indicating strong concordance between the assembly and the underlying sequencing data.

Expression-weighted contiguity was examined to assess transcript quality among biologically relevant genes, revealing a gene-level Ex95 N50 of 1,570 bp, indicating that the most highly expressed 95% of genes are represented by relatively long and contiguous transcripts, while inclusion of lowly expressed genes led to a reduction in N50 values. Together, these metrics support the robustness and biological relevance of the assembled transcriptome.

Functional annotation of the transcriptome using Trinotate resulted in the assignment of putative functional information to a substantial fraction of transcripts. Swiss-Prot similarity searches identified significant BLASTX hits for 31,790 transcripts, while BLASTP searches annotated 27,278 predicted proteins. In addition, Pfam domain searches identified conserved protein domains in 29,151 predicted proteins. Collectively, these annotation results provide a solid functional framework for downstream expression and enrichment analyses.

### Comparative differential gene expression analysis reveals developmentally structured transcriptional profiles

**Fig. 2:**
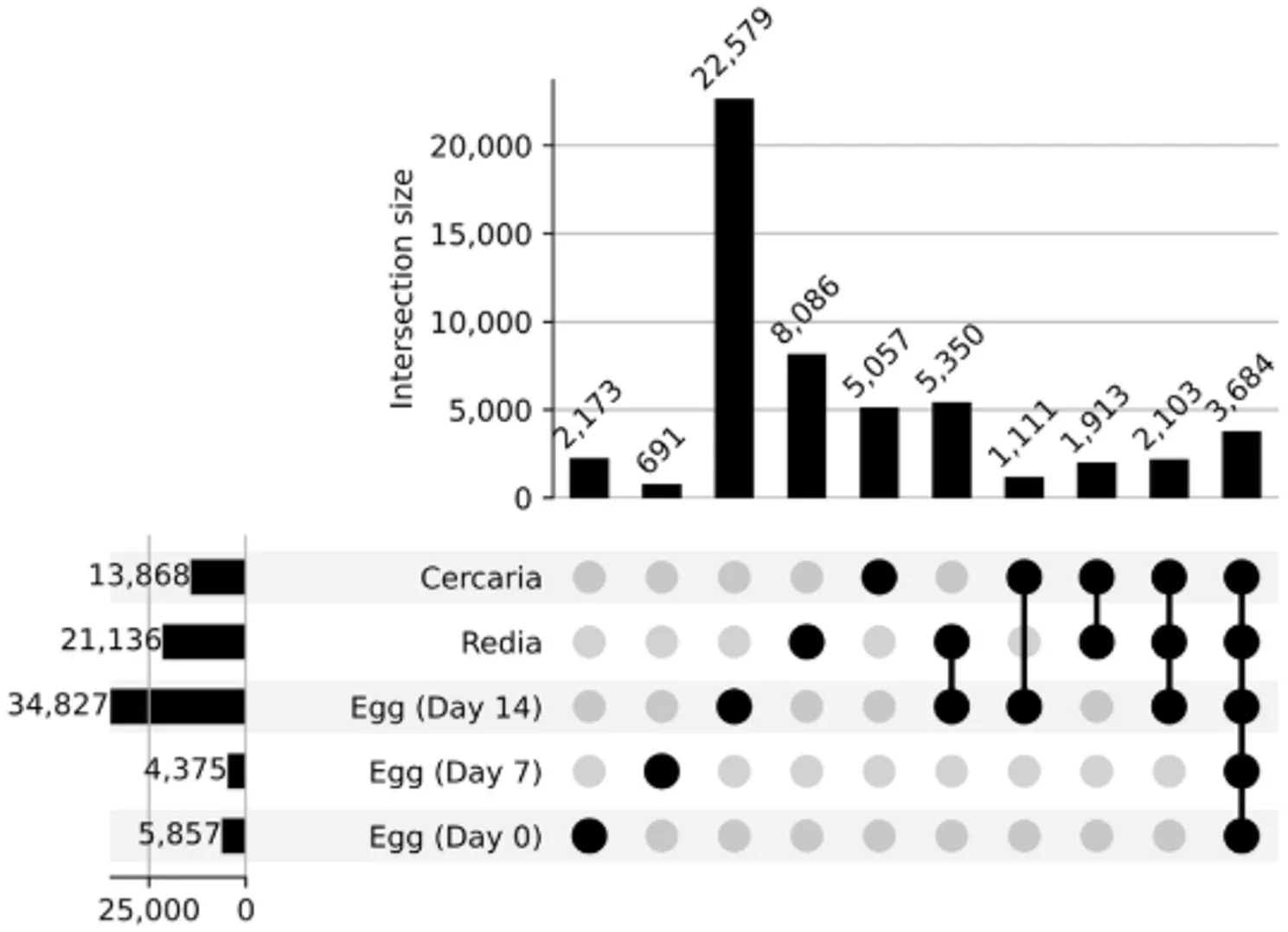
UpSet plot showing the top 10 intersections of expressed genes across the lifecycle stages (T0, T7, T14, Redia, and Cercaria). Genes were considered expressed if they showed TPM ≥ 1 in at least two replicates. The vertical bars indicate the size of each intersection (number of genes shared by the indicated combination of stages), while the connected black dots below denote the lifecycle stages contributing to each intersection. Horizontal bars on the left represent the total number of expressed genes per life stage. The complete intersection matrix for all five lifecycle stages is provided in Supplementary Fig. S1

The intersection analysis investigates the extent of shared versus stage-specific expression across the different developmental stages of *C. daubneyi*, to identify conserved core transcriptional programs and stage-specific molecular signatures (Fig. 2, Supplementary Fig. S1). A total of 3,684 genes were expressed consistently across all five life stages. The largest intersection comprised 22,579 genes uniquely expressed in the eye-spot stage. Generally, the number of expressed genes varied between stages, with the eye-spot stage showing the highest total number, followed by rediae. Early egg stages had the lowest number of expressed genes. Notably, the intersections between each different egg stages fall outside the ten largest intersections displayed in Fig. 2 and are therefore not shown. The complete intersection matrix across all five lifecycle stages is provided in Supplementary Fig. S1. Additionally, Supplementary Table 1 provides the per-sample summary statistics including total reads processed, total reads mapped, mapping rate, and number of genes detected at TPM ≥ 1 for all 15 samples.

**Fig. 3:**
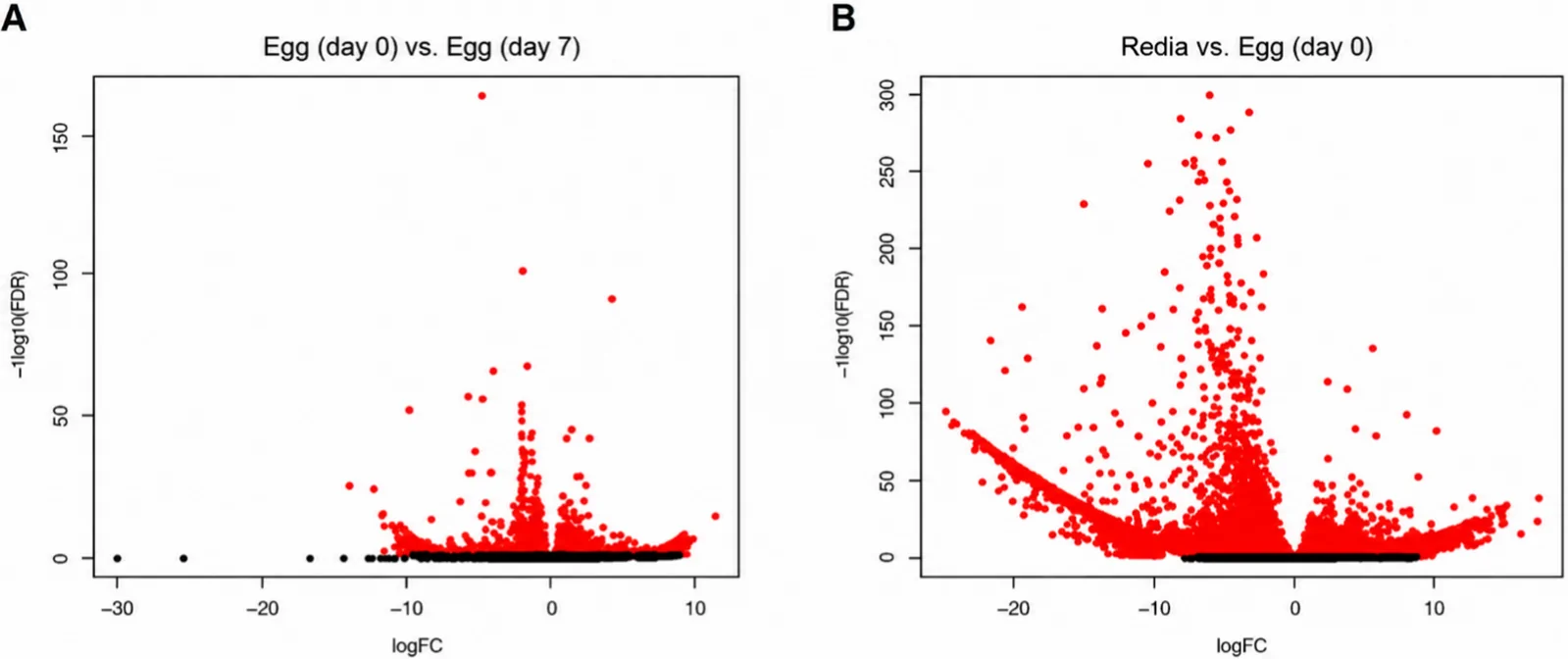
Volcano plots of pairwise differential gene expression analysis between lifecycle stages *of C. daubneyi*. X-axis represents the log_2_ Fold Change and the y-axis shows the -log_10_ of the False Discovery Rate (FDR). Each dot depicts a gene. Genes with significant up or down regulation (FDR ≤ 0,05 ) are colored in red. **A** Egg (Day 7) over Egg (Day 0). **B** Egg (Day 0) over Redia

To quantify transcriptional changes associated with developmental progression, pairwise differential gene expression analyses were performed between all life-cycle stages (Fig. 3, Supplementary Fig. S2).

The smallest differences in gene expression were observed between the freshly excreted and developing egg groups, with only a limited number of genes being significantly up- or downregulated (Fig. 3A). This indicates that freshly excreted eggs (T0) and eggs at the onset of development (T7) display highly similar transcriptional profiles. In contrast, the transition to the eyespot stage (T14) was characterized by substantial transcriptional changes: in the redia versus freshly excreted egg comparison, numerous genes were significantly up- as well as downregulated, accompanied by an overall increase in transcriptional activity (Fig. 3B).

Marked expression differences were also evident when rediae were compared with all egg stages, underscoring the major transcriptional shift associated with this developmental transition.

As expected a general pattern emerged: the closer two developmental stages were in time, the fewer genes exhibited expression changes and/or the less significant these changes were. Conversely, comparisons of more distant stages revealed more extensive and statistically robust transcriptional differences. In particular, the early stage eggs (T0 and T7) showed highly similar expression profiles, whereas later developmental contrasts revealed progressively larger and more significant shifts in gene activity.

**Fig. 4:**
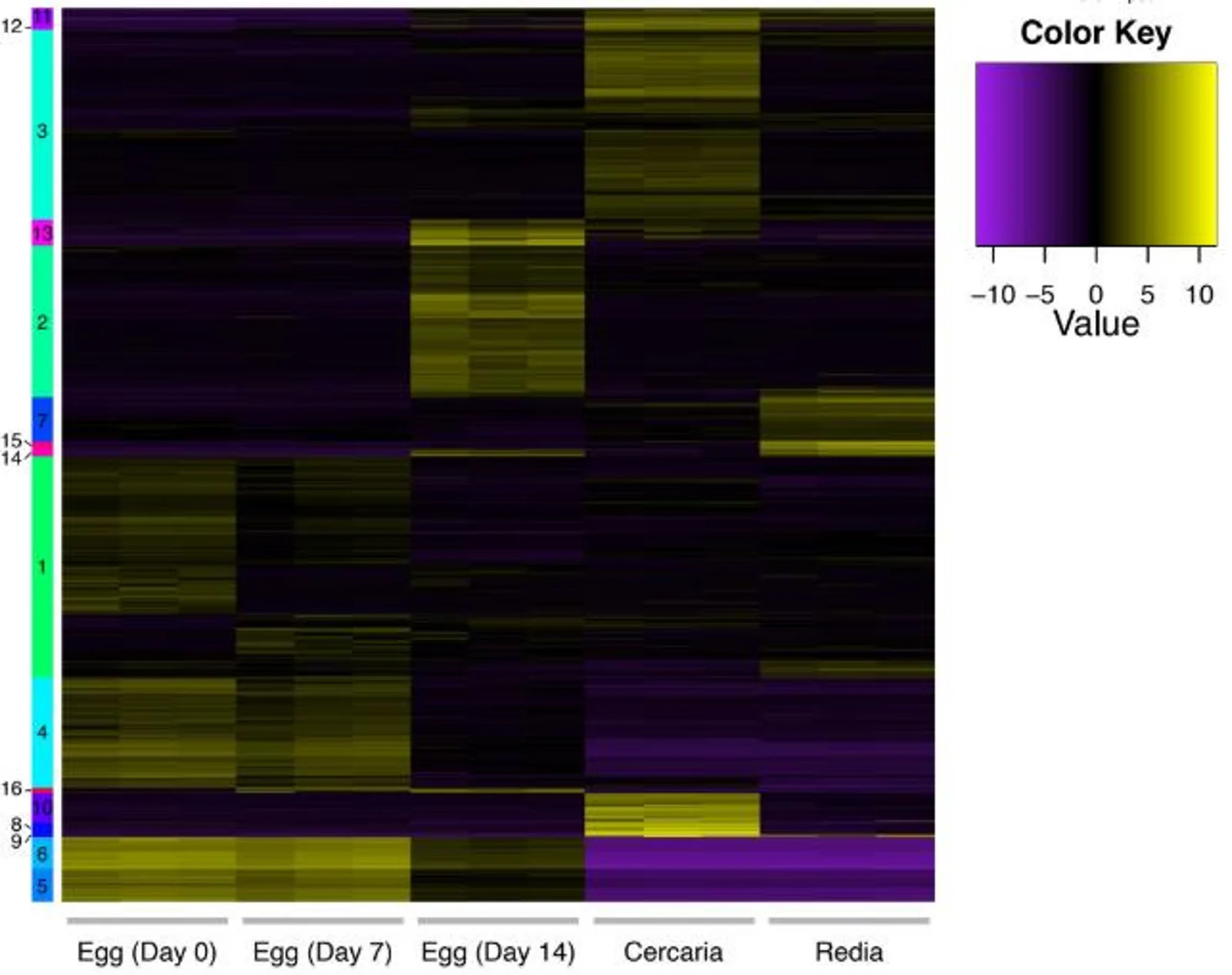
Hierarchical clustering heatmap of differentially expressed genes across the *C. daubneyi* developmental stages. For each pairwise comparison, up to 1,000 genes meeting the differential-expression thresholds (FDR < 0.001 and log₂ fold change > 2) were retained; the union of these gene sets was used for clustering. Expression values shown are log₂-transformed, normalised and median-centred per gene. The colour scale represents relative expression levels: yellow indicates expression higher than the gene’s cross-sample median, purple indicates expression lower than the median and black values correspond to expression close to the median. Colums represent the three biological replicates of each developmental stage. Genes (rows) were grouped by hierarchical clustering to reveal modules of co-regulated transcripts with similar expression profiles across developmental stages. The coloured side bar indicates the 16 subclusters obtained by cutting the gene dendrogram at 30% of its height (--Ptree 30, see Methods). These subclusters served as the input for the subsequent GO enrichment analyses presented in Figs. 5 and 6

The heat map of differentially expressed genes revealed a clear separation of the *C. daubneyi* developmental stages at the transcriptional level (Fig. 4). Hierarchical clustering grouped samples according to their developmental phase with biological replicates clustering closely together, indicating high reproducibility. Each stage exhibited a distinct expression profile, reflecting stage-specific biological processes. In particular, cercaria and the later and the eyespot egg stage showed strongly divergent expression patterns compared to earlier stages. The data demonstrates dynamic and stage specific regulation of gene expression across the parasite’s life cycle.

### Functional annotation and GO-enrichment analysis indicate *C. daubneyis* capability to adapt to environmental and developmental changes

To gain insight into the biological processes underlying the stage-specific transcriptional programs, we performed Gene Ontology (GO) enrichment analyses on selected gene expression subclusters exhibiting clear developmental stage associations. Enriched subclusters were analyzed in more detail. Additionally, Supplementary Tables 2 and Supplementary Figure S3 provide the complete enrichment results and expression profiles for all subclusters.

**Fig. 5:**
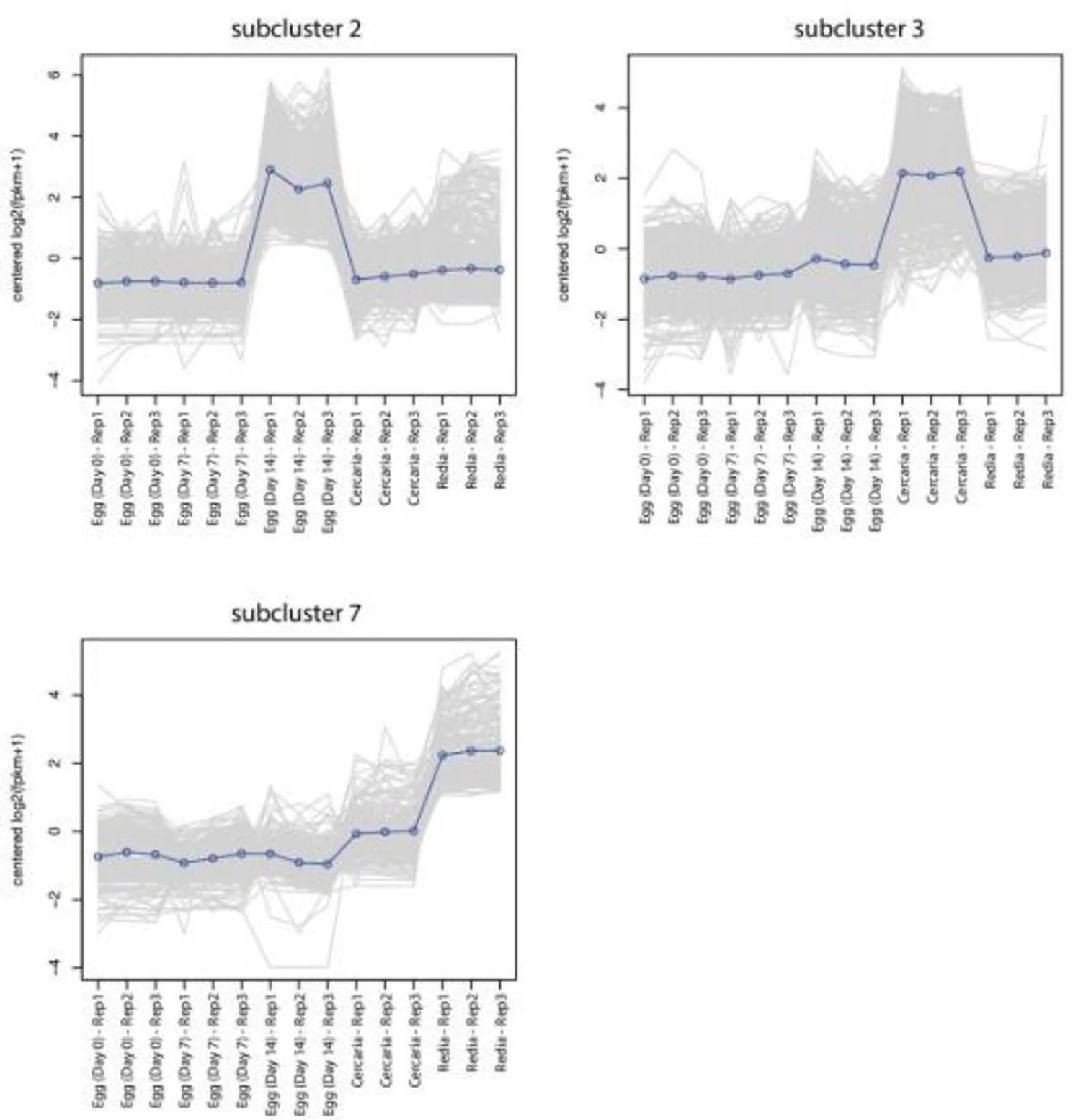
Subcluster 2 (representing eye-spot-eggs T14), 3 (representing cercariae) and 7 (representing rediae). X-axis: replicates of the groups. Y-axis: log-transformed, median-centered FPKM values corresponding to changes in gene expression. Blue line represents the mean line, grey lines reflect individual genes. Each graph shows an upregulation of gene expression in only one developmental group. These genes were used for subsequent GO enrichment analysis (see supplementary table 6). Supplementary Fig. S3 shows the expression profiles for all 16 subclusters

Enriched GO terms were identified for subclusters corresponding to eye-spot stage (subcluster 2), redial stage (subcluster 7) and cercarial stage (subcluster 3), revealing distinct functional signatures associated with each developmental context (Fig. 5).

**Fig. 6:**
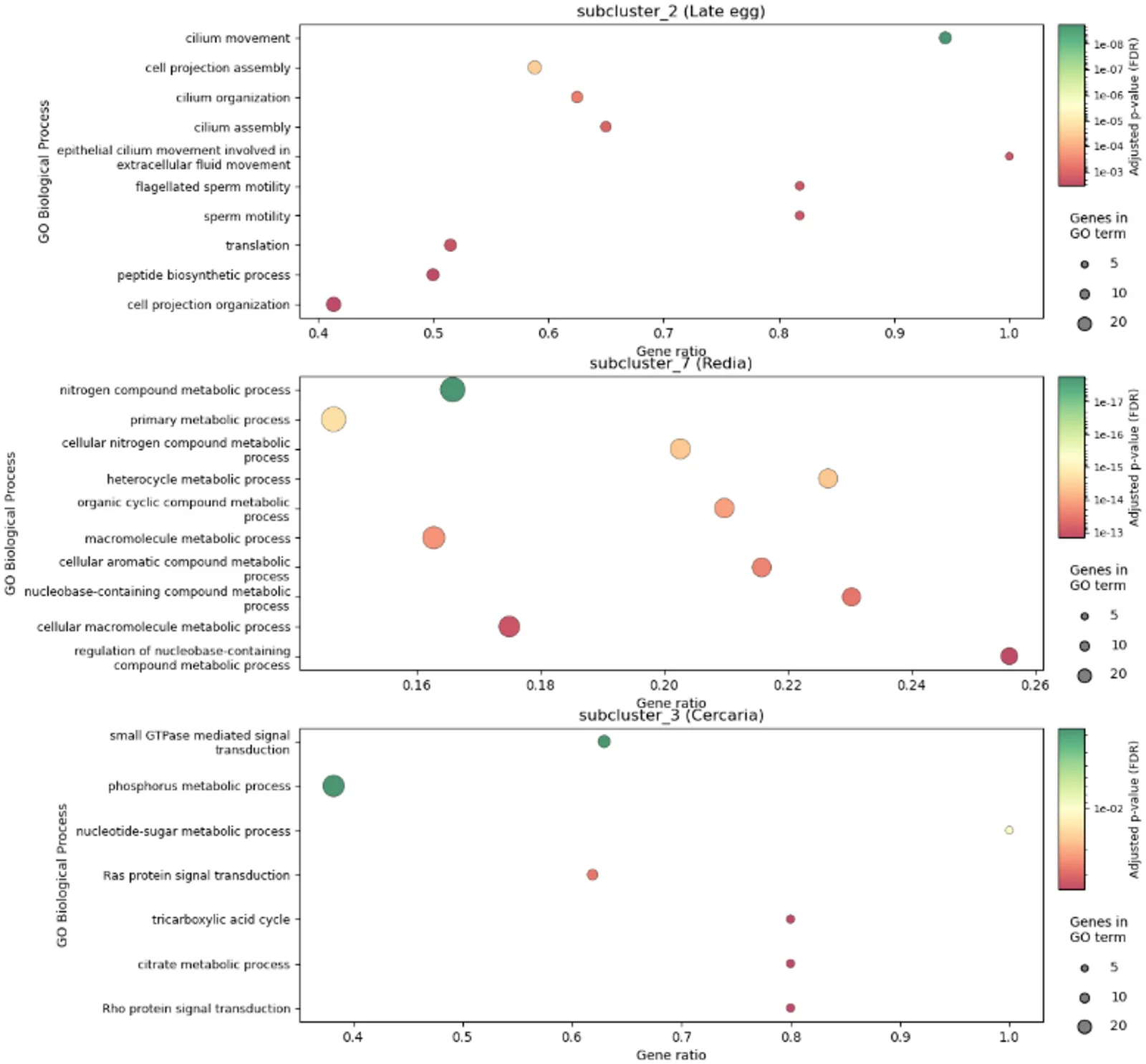
GO enrichment analysis (Biological Process) of the three identified subclusters (subcluster_2: Late egg; subcluster_7: Redia; subcluster_3: Cercaria). Shown are significantly enriched GO terms for each subcluster. The x-axis indicates the gene ratio (the proportion of genes within a subcluster associated with a given GO term), point size represents the number of genes per GO term, and color denotes the adjusted p-value (FDR)

Genes in subcluster 2 (eye-spot staged eggs) were significantly enriched for cilium- and motility- related biological processes, including cilium movement, organization and assembly, as well as sperm motility. In addition, terms related to translation and peptide biosynthetic processes were overrepresented, indicating active protein synthesis during this developmental stage. Key marker genes driving the cilium movement GO term enrichment (GO:0003341, FDR = 1.76e-09) in subcluster 2 are listed in Supplementary Table 3 .These include multiple structural and regulatory components of the axonemal machinery, such as outer dynein arm-docking complex subunits 2, 3 and 4, dynein axonemal heavy chain 6, dynein regulatory complex subunit 4, tektins 1, 2 and 4, sperm-associated antigens 6 and 17, radial spoke head protein 9, cilia- and flagella-associated protein 45, calaxin, and sperm flagellar protein 1. In subcluster 7 (rediae), genes were predominantly associated with a wide range of metabolic processes, including nitrogen compound metabolism and macromolecule turnover. Additional enrichment was observed for regulatory processes associated with nucleobase-containing compounds. Subcluster 3 (cercariae) was characterized by genes involved in signal transduction, including GTPase-mediated signaling as well as Ras and Rho protein signal transduction. Furthermore, metabolic pathways relating to energy metabolism such as the tricarboxylic acid cycle and citrate metabolic processes were represented (Fig. 6).

## Discussion

The heteroxenous life cycle of *C. daubneyi* is extremely complex and involves many different developmental stages. In the past, transcriptome studies on the mammalian life cycle stages and a genome study have already been successfully conducted [28, 29, 44]. However, with regard to the epidemiological question of how the parasite behaves in the environment and in the intermediate host, the developmental stages outside the final host are also particularly important. Such data for *C. daubneyi* have been completely lacking until now.

In this study we created a transcriptome dataset incorporating environmental and intermediate host stages of *C. daubneyi*. Our assembly N50 (1,286 bp) is similar to the one obtained by Huson et al. (2021b) using long-read sequencing (1,128 bp) when analyzing the mammalian host life stages of *C. daubneyi*. In addition, the N50 size is also consistent with other helminth transcriptomes such as *Schistosoma turkestanikum* (1480 bp) *or Fasciola gigantica* (1520 bp) [45, 46], supporting the good quality of the transcriptome assembly obtained here.

The recently published *C. daubneyi* genome [44] provides a valuable reference for the species; however, its annotation was generated without transcriptomic data from environmental and intermediate host stages and may therefore not fully capture the transcripts expressed during these previously unsampled phases of the life cycle. To assess this, we compared our Trinity-assembled transcripts with the genome-derived transcriptome by BLAST (e-value < 1e-5 ). Of the 47,464 Trinity genes that were differentially expressed in at least one pairwise stage comparison, 38,270 (approx. 81%) had no similar sequence in the current genome annotation. Furthermore, among the top 1,000 most differentially expressed genes in at least one pairwise comparison (3,735 Trinity genes), 1,528 (~ 41%) were absent from the genome annotation. Manual inspection of a subset of these transcripts confirmed that they are present in the underlying genome sequence but not represented in the annotation, indicating that these are genuine coding sequences missed during genome annotation rather than Trinity assembly artefacts. Relying on the current genome annotation alone would therefore have excluded a substantial fraction of the biologically relevant signal from our differential expression and enrichment analyses. A comparable situation has been reported for *F. hepatica*, where transcriptome profiling of intra-snail stages identified 1,744 previously unannotated transcripts, most of them stage specific for miracidial and intra-molluscan phases [47]. To provide a resource that complements the genome annotation, gene-level normalized read counts obtained by mapping all samples against the current *C. daubneyi* genome annotation are additionally provided as Supplementary Table 4.

The differential expression analysis revealed pronounced transcriptional changes across the different study groups within a statistically significant range. Hierarchical clustering delineated distinct and interpretable clusters, each corresponding to developmental phases characterized by specific patterns of transcriptional regulation. These patterns were consistently reproducible across biological replicates.

The relatively small set of genes expressed across all developmental stages suggests that only a limited core transcriptome is maintained throughout development. Genes detected throughout all three egg timepoints but absent from rediae and cercariae (Suppl. Figure 1) likely represent a core egg-stage transcriptome sustained throughout embryonation. During early egg development, under stable environmental conditions, relatively few specific genes appear to be required, as no major transcriptional changes are observed here and the exclusive pairwise intersections between adjacent egg stages (T0-T7 and T7-T14) remain comparatively small. The transition to the redial and cercarial stages involves considerably more transcriptional changes and therefore appears to be more challenging for the trematode, with a comparably high number of uniquely expressed genes in these two lifecycle stages.

Finally, the substantial increase in the T14-exclusive intersection reflects a temporally restricted transcriptional program associated with miracidial assembly. The transition from eggs to free-living miracidia entails particularly pronounced molecular adaptations and regulation on the transcriptomic level to ensure the parasite’s survival and successful host finding under the fluctuating environmental conditions at this stage. Similar observations have previously been reported when comparing transcripts of *F. gigantica* eggs and miracidia (comparable to our eye-spot-stage). Miracidia exhibited nearly three times the number of uniquely expressed transcripts compared to early eggs [45].

The GO enrichment analysis revealed substantial functional differences among the subclusters, indicating clear stage-specific biological programs, as previously described for *F. gigantica* [45]. In the life cycle of *C. daubneyi*, the initially unembryonated eggs continue their development only after being released into suitable environmental conditions, with moisture and temperature acting as key determinants [13]. Genes associated with the eye-spot subcluster (Subcluster 2) were strongly enriched for processes related to ciliary and flagellar structure and motility including cilium assembly, movement and sperm motility. Cilia are present on a wide range of cell types and generate motility. Their internal skeleton, the axoneme, consists of a characteristic “9 + 2” arrangement of microtubules, where dynein motor proteins provide the force required for movement [48]. The miracidia of *C. daubneyi* are equipped with numerous motile cilia that enable directed swimming toward the intermediate host, which is essential for penetration [14]. Proteomic studies in *Schistosoma mansoni* miracidia similarly demonstrated upregulation of ciliary dynein arms, most likely linked to flagellar motility [49]. The strong upregulation of cilia- and dynein-related transcripts in these eyespot-eggs therefore provides compelling evidence for successful progression to the motile miracidial stage ready for host-seeking. The coherence of the axonemal marker gene set further supports a genuine biological signal rather than a technical artifact. The enrichment is driven by multiple distinct structural and regulatory components of the same biological machinery — outer dynein arm-docking complex subunits, dynein heavy chains, tektins, radial spoke proteins, and flagella-associated proteins — coordinately upregulated in the same stage-specific subcluster. This pattern is inconsistent with annotation bias, which would be expected to affect all stages equally rather than concentrate in a single stage-specific subcluster, or with transcriptome fragmentation artifacts, which typically manifest as multiple Trinity components with nearly identical rather than functionally distinct annotations.

Comparable findings have also been reported during transcriptomic analyses of newly migrated and adult *C. daubneyi*, where dynein upregulation was attributed to trematode spermatozoa [28]. Indeed, dynein forms part of the axonemal structure of trematode sperm cells [50], and is therefore consistently expressed in sexually reproducing adults [28]. In Subcluster 2, several significantly enriched GO terms likewise pointed to sperm-related cilia and their motility. While the development from unembryonated egg to motile miracidium proceeds asexually through cell division and differentiation, the presence of germline precursors within the mature miracidium [14] may explain this overlap. More plausibly, however, the shared enrichment reflects conserved motility mechanisms: miracidial swimming closely resembles that of sperm, both relying on the axonemal microtubule structure and dynein-driven propulsion [50]. These mechanisms are evolutionarily conserved across diverse cell types and function independently of reproduction, providing a versatile means of cellular locomotion.

The redia is a highly active, quickly developing larval stage in the intermediate snail host. The observed significant upregulation of general metabolic and biosynthetic processes within the GO-Enrichment analysis of Subcluster 7, reflects the high demand of rediae for energy- and building blocks, associated with growth, replication and asexual proliferation inside the host. To date, Zhang et al. [45] represent the only study to perform a detailed transcriptomic analysis of rediae from a trematode species closely related to *C. daubneyi*, namely *F. gigantica*. Similar to this study, in *F. gigantica*, numerous genes associated with metabolic processes, transcription, translation and repair were highly upregulated during redial stage. These findings indicate that *C. daubneyi* rediae meet the substantial biological demands of clonal expansion within the snail host by modulating transcriptional activity and biosynthetic capacity of essential processes.

For the transcriptomic analysis of *C. daubneyi*, only fully mature, motile cercariae ready for environmental encystment were collected from snails and all pre-cercarial stages were excluded. Analysis of Subcluster 3, which comprised genes significantly upregulated exclusively in cercariae, revealed particularly high in signal transduction and energy metabolism with a high gene activity in the tricarboxylic acid (TCA) cycle, citrate metabolism and the electron transport chain.

The activation of signal transduction pathways indicates enhanced environmental sensing and responsiveness. Motile *C. daubneyi* cercariae aim to reach nearby plants and encyst as infectious metacercariae following tail loss [13]. Since infection of the definitive host occurs primarily through ingestion of metacercariae-contaminated vegetation, cercarial targeting of plants is critical for transmission [15]. Like miracidia, trematode cercariae possess limited energy reserves and must reach their targets rapidly. We propose, as a testable hypothesis, that the upregulation of signal transduction pathways in cercariae reflects active environmental sensing and cytoskeletal reorganization during host-seeking and encystment. Supporting this, the genes driving the enrichment of small GTPase mediated signal transduction (GO:0007264), Ras protein signal transduction (GO:0007265) and Rho protein signal transduction (GO:0007266) in Subcluster 3 encode a coherent set of candidates including members of the Rho/Ras/Rab GTPase family (RhoC, Rac1, Rac2, Ral-B, Rab6, Rab-3, RHO4), key regulatory proteins (Rho GDP-dissociation inhibitor 1, Rho GTPase-activating protein 1, the guanine nucleotide exchange factor PLEKHG5), and the downstream effector kinase PAK1 — a canonical target of Rac and Cdc42 signaling (Supplementary Table 5). Future functional studies, such as pharmacological inhibition of Rho/Rac GTPase signaling in cercariae coupled with assessment of encystment efficiency, could directly test this hypothesis.

The TCA cycle is a central mitochondrial metabolic pathway that provides cellular energy under aerobic conditions and is indirectly dependent on oxygen, which is required for energy production via the respiratory chain. Huson et al. [28] also discussed the TCA cycle in *C. daubneyi* transcriptomes of mammalian host stages. Their analysis showed that TCA cycle components were most strongly expressed in newly excysted juveniles (NEJs) and gradually downregulated as the parasite matured to the adult stage. Concurrently, genes associated with the malate dismutation pathway, an anaerobic energy-generating mechanism, were upregulated. While NEJs still have some access to oxygen, adult parasites in the anaerobic rumen do not. Huson et al. [28] therefore hypothesized a developmental shift in energy metabolism from the aerobic TCA cycle to the anaerobic malate dismutation pathway. Similarly, Cwiklinski et al. (2015) demonstrated in *F. hepatica* that parasite growth restricts oxygen diffusion into tissues, driving a transition from aerobic TCA metabolism to anaerobic malate dismutation. A comparable ecological gradient of oxygen availability is encountered throughout the rest of the *C. daubneyi* life cycle: free-living miracidia and cercariae inhabit well-oxygenated freshwater microenvironments, where dissolved oxygen typically approaches atmospheric saturation. Within the infected and stressed intermediate snail host, by contrast, oxygen availability is considerably more restricted [51]. These parts of the *C. daubneyi* life cycle therefore also traverses a stepwise gradient from fully aerobic external environments through a hypoxic intramolluscan phase. The present observation that cercariae show upregulation of genes for the TCA cycle, electron transport chain and citrate metabolism supports Huson et al.’s [28] findings and indicates that also *C. daubneyi* cercariae exploit aerobic energy production in their environment.

## Conclusions

As research on other trematode species has demonstrated the value of “-omics” approaches, this study similarly leveraged the advantages of RNA-seq to investigate the previously understudied trematode *C. daubneyi* [2]. For the first time, transcriptomic data were generated and analyzed for selected developmental stages of *C. daubneyi* in the environment and within the snail intermediate host. These analyses revealed that *C. daubneyi* exhibits stage-specific transcriptional adaptations during the examined life phases, enabling the parasite to respond to its developmental tasks or its environmental and intermediate host conditions.

Our results demonstrate that *C. daubneyi* exhibits stage-specific molecular adaptations at each life stage that are critical for survival, development and or environmental interaction. Targeted investigation of these stage-specific genes, such as cilia-related genes in eyespot-stage eggs crucial for host finding, metabolic process regulation needed for successful proliferation in rediae or energy metabolism genes required for survival in the environment in cercariae, provides opportunities to develop intervention strategies that disrupt the parasites life cycle progression and thereby effectively support the control of Paramphistomidosis in the future.

To achieve a more comprehensive understanding of the parasite’s life cycle, analysis of additional developmental stages—particularly miracidia, sporocysts and metacercariae—would be desirable. All three stages are critical for sustaining the life cycle and for successful infection of the intermediate and definitive hosts. In particular, a direct transcriptomic comparison between the infective miracidium and the first intramolluscan developmental stage, the mother sporocyst, could provide valuable insights into key developmentally regulated genes governing the transition from the environmental to the intramolluscan phase and thereby identify potential targets for transmission control. Similarly, investigating and comparing freshly excreted and fully encysted metacercariae may reveal key genes crucial for transmission and surviving in the environment. The results of this study suggest that these stages may also express stage-specific genes, the investigation of which could reveal additional targets for control strategies. Integrating complementary “-omic” approaches, including secretomics and proteomics across all developmental stages could further elucidate mechanisms of host invasion, environmental resilience and metabolic adaptation. The transcriptomic dataset generated here further provides a valuable resource for future re-annotation of the *C. daubneyi* genome. Our comparative BLAST analysis indicated, that a substantial fraction of the transcripts differentially expressed during the environmental and intermediate host stages is not represented int the current genome annotation. Integrating these transcriptomic data into an updated genome annotation would therefore be a next step towards a more complete reference for future comparative and functional studies of *C. daubneyi*.

The findings underscore the importance of molecular adaptation strategies in trematode development and represent a step toward identifying novel interventions that could disrupt the *C. daubneyi* life cycle, ultimately reducing reinfection rates and limiting the emerging impact on livestock health.

## Supplementary Information


Supplementary Material 1. Supplementary Table 1: Provides per-sample summary statistics including total reads processed, total reads mapped, mapping rate, and number of genes detected at TPM ≥ 1 for all 15 samples.



Supplementary Material 2. Supplementary Table 5: Provides candidate genes driving the small GTPase, Ras and Rho protein signal transduction GO term enrichment in Subcluster 3.



Supplementary Material 3. Supplementary Fig. S2: Volcano plots of pairwise differential gene expression analysis between lifecycle stages of C. daubneyi. X-axis represents the log2 Fold Change and the y-axis shows the -log10 of the False Discovery Rate (FDR. Each dot depicts a gene. Genes with significant up or down regulation (FDR ≤0,05 ) are colored in red.



Supplementary Material 4. Supplementary Table 6: Provides a complete list of significantly enriched GO terms, including the associated gene identifiers, gene ratios, *p*-values and FDR values.



Supplementary Material 5. Supplementary Table 3: Provides key marker genes driving the cilium movement GO term enrichment (GO:0003341, FDR = 1.76e-09) in subcluster 2.



Supplementary Material 6. Supplementary Table 2: Provides the complete GOseq BP enrichment results for all subclusters, including all GO terms tested and their associated FDR values, allowing readers to assess the enrichment landscape across all clusters.



Supplementary Material 7. Supplementary Fig. 1: UpSet plot showing the intersections of expressed genes across all five lifecycle stages (T0, T7, T14, Redia, and Cercaria). Genes were considered expressed if they showed TPM ≥ 1 in at least two replicates. The vertical bars indicate the size of each intersection (number of genes shared by the indicated combination of stages), while the connected black dots below denote the life stages contributing to each intersection. Horizontal bars on the left represent the total number of expressed genes per lifecycle stage.



Supplementary Material 8. Supplementary Fig. S3: Expression profiles of all 16 subclusters. X-axis: replicates of the groups. Y-axis: log-transformed, median-centered FPKM values corresponding to changes in gene expression. Blue line represents the mean line, grey lines reflect individual genes.



Supplementary Material 9. Supplementary Table 4: Provides gene-level normalized read counts obtained by mapping all samples against the current C. daubneyi genome annotation.


## Data Availability

The sequencing data generated for this project have been deposited in the European Nucleotide Archive and is accessible through ENA study accession number PRJEB108824 (https://www.ebi.ac.uk/ena/browser/view/PRJEB108824).

## Abbreviations

GO: Gene Ontology
NaCl: Sodium Chloride
PCR: Polynucleotide Chain Reaction
SBS: Sequencing-By-Synthesis
ORF: Open Reading Frame
FDR: False Discovery Rate
TPM: Transcripts Per Million
TCA: Tricarboxylic Acid Cycle
NEJ: Newly Excysted Juvenile
