# Supplementary material for: Transcriptomic analysis of eggs, rediae and cercariae reveal stage-specific adaptations in the rumen fluke Calicophoron daubneyi
Source: BMC Genomics. 2026 Sep 2;27:726. doi: 10.1186/s12864-026-13271-z (PMC13536829; doi:10.1186/s12864-026-13271-z)
Supplement: Supplementary file 1 [file 12864_2026_13271_MOESM3_ESM.pdf]

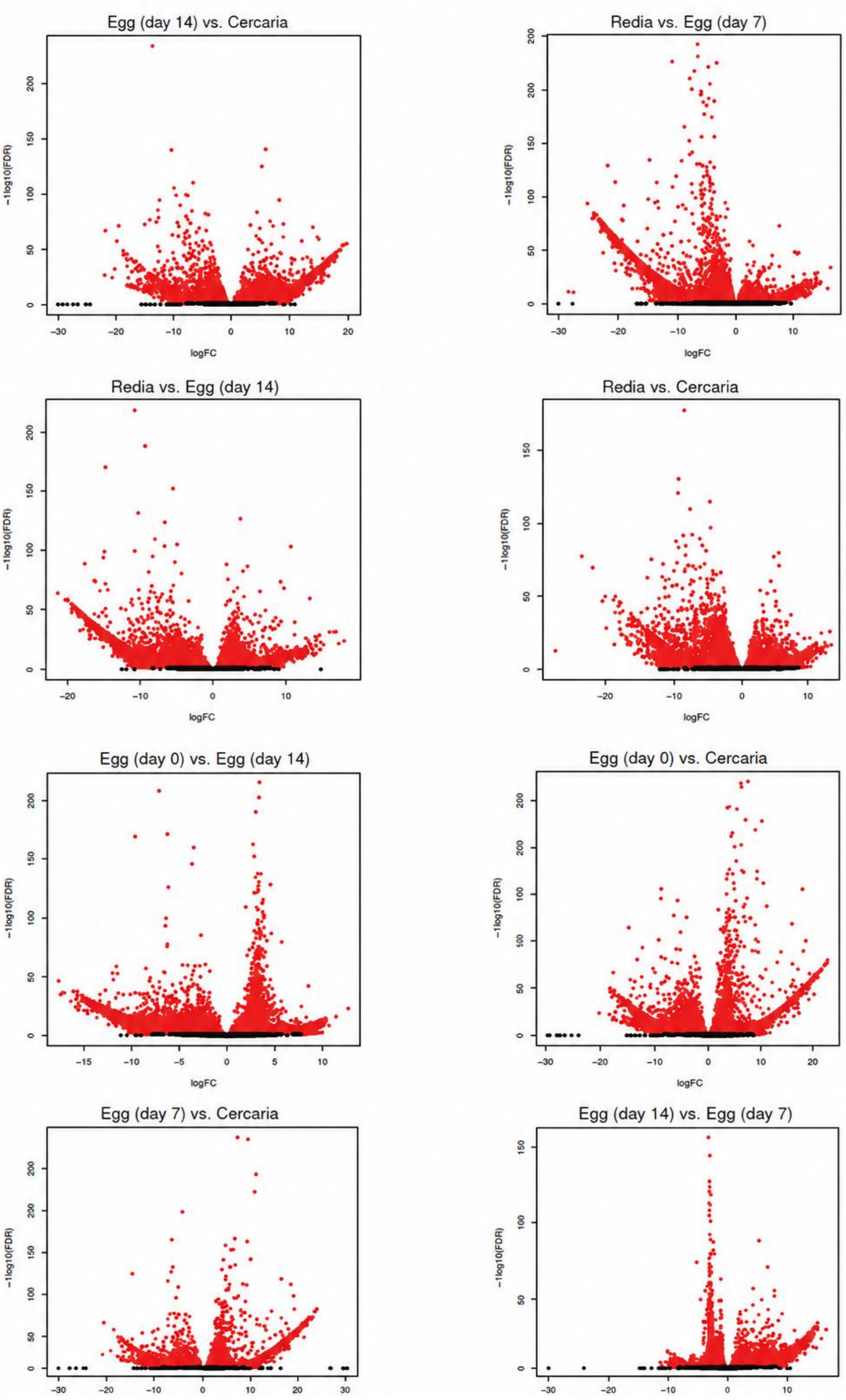

Supplementary Fig. S2: Volcano plots of pairwise differential gene expression analysis between lifecycle stages of *C. daubneyi*. X-axis represents the $\log_2$ Fold Change and the y-axis shows the -$\log_{10}$ of the False Discovery Rate (FDR). Each dot depicts a gene. Genes with significant up or down regulation (FDR ≤0,05) are colored in red.
