# Supplementary material for: Transcriptomic analysis of eggs, rediae and cercariae reveal stage-specific adaptations in the rumen fluke Calicophoron daubneyi
Source: BMC Genomics. 2026 Sep 2;27:726. doi: 10.1186/s12864-026-13271-z (PMC13536829; doi:10.1186/s12864-026-13271-z)
Supplement: Supplementary file 2 [file 12864_2026_13271_MOESM7_ESM.pdf]

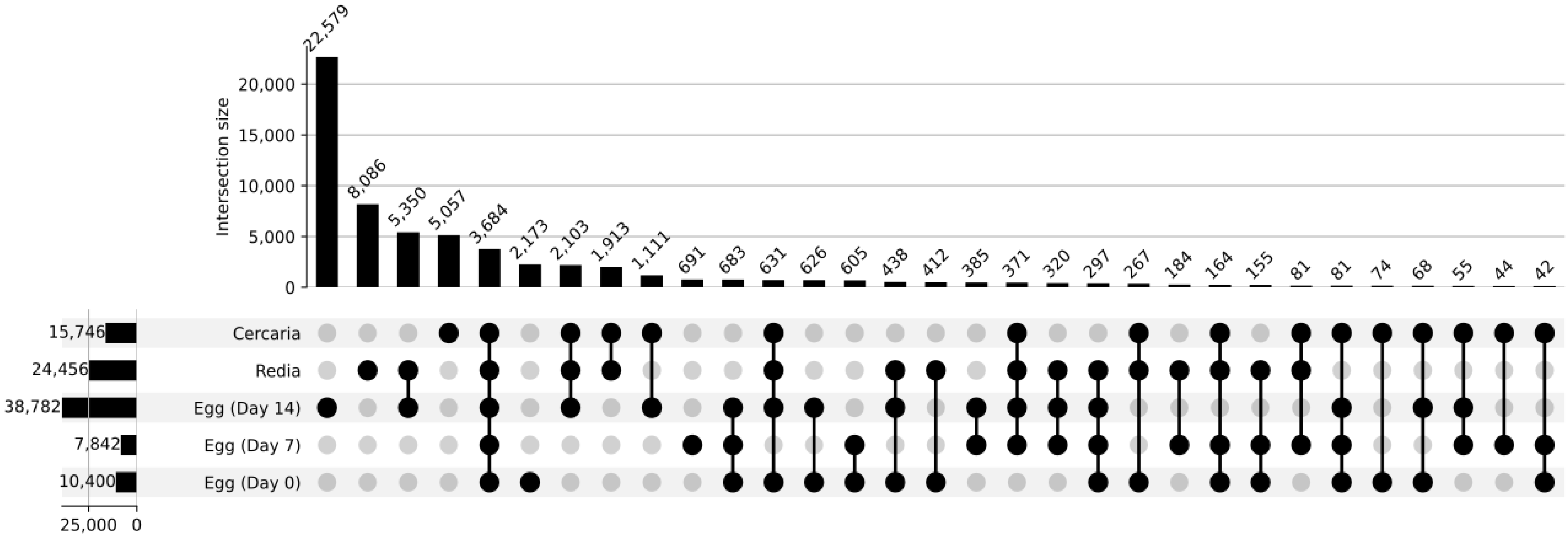

Supplementary Fig. S1: UpSet plot showing the intersections of expressed genes across all five lifecycle stages (T0, T7, T14, Redia, and Cercaria). Genes were considered expressed if they showed TPM ≥ 1 in at least two replicates. The vertical bars indicate the size of each intersection (number of genes shared by the indicated combination of stages), while the connected black dots below denote the life stages contributing to each intersection. Horizontal bars on the left represent the total number of expressed genes per lifecycle stage.
