# Supplementary material for: Transcriptomic analysis of eggs, rediae and cercariae reveal stage-specific adaptations in the rumen fluke Calicophoron daubneyi
Source: BMC Genomics. 2026 Sep 2;27:726. doi: 10.1186/s12864-026-13271-z (PMC13536829; doi:10.1186/s12864-026-13271-z)
Supplement: Supplementary file 3 [file 12864_2026_13271_MOESM8_ESM.pdf]

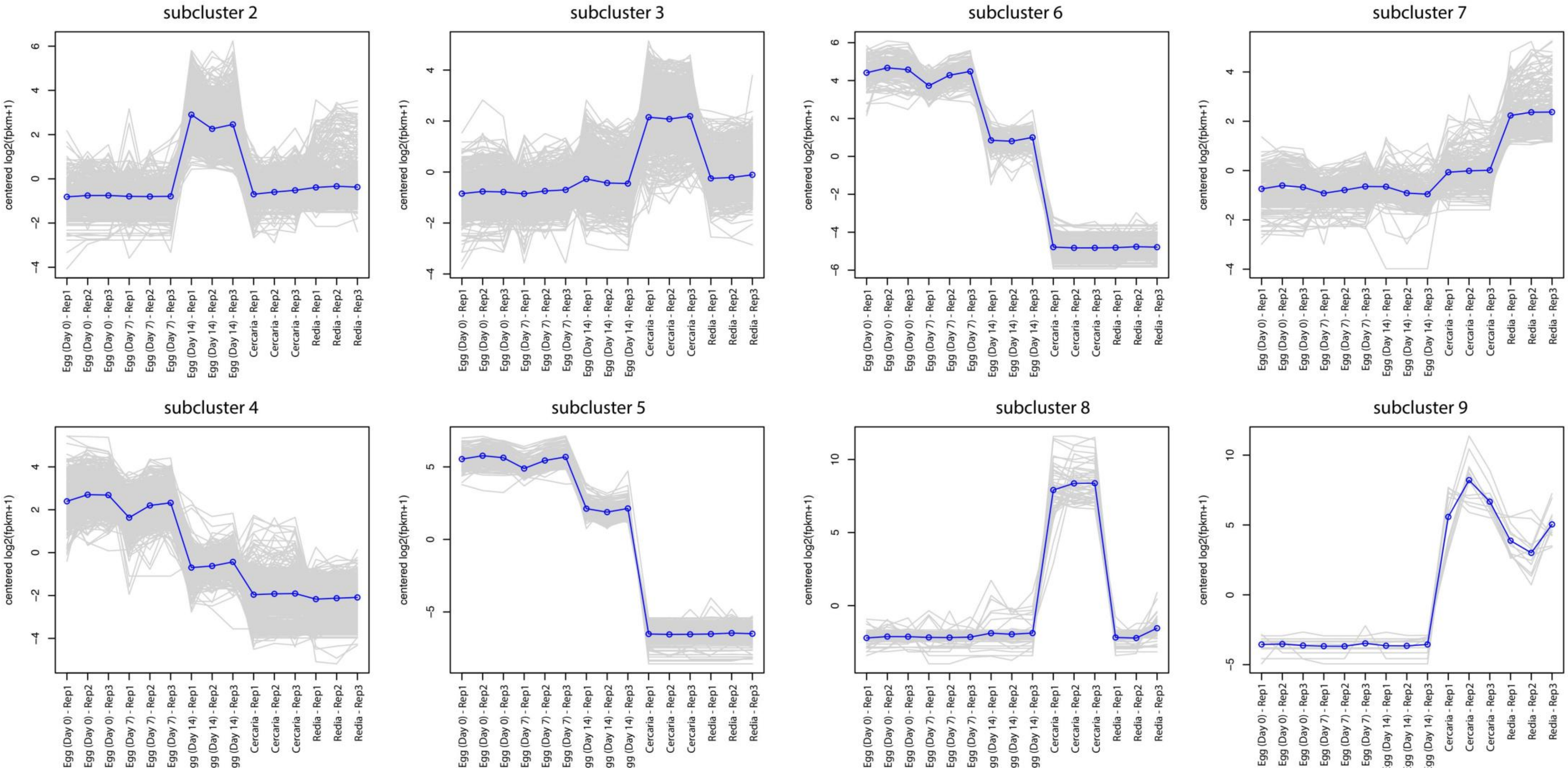

subcluster 2
subcluster 3
subcluster 6
subcluster 7
subcluster 4
subcluster 5
subcluster 8
subcluster 9
centered log2(fpkm+1)
Egg (Day 0) - Rep1
Egg (Day 0) - Rep2
Egg (Day 0) - Rep3
Egg (Day 7) - Rep1
Egg (Day 7) - Rep2
Egg (Day 7) - Rep3
Egg (Day 14) - Rep1
Egg (Day 14) - Rep2
Egg (Day 14) - Rep3
Cercaria - Rep1
Cercaria - Rep2
Cercaria - Rep3
Redia - Rep1
Redia - Rep2
Redia - Rep3

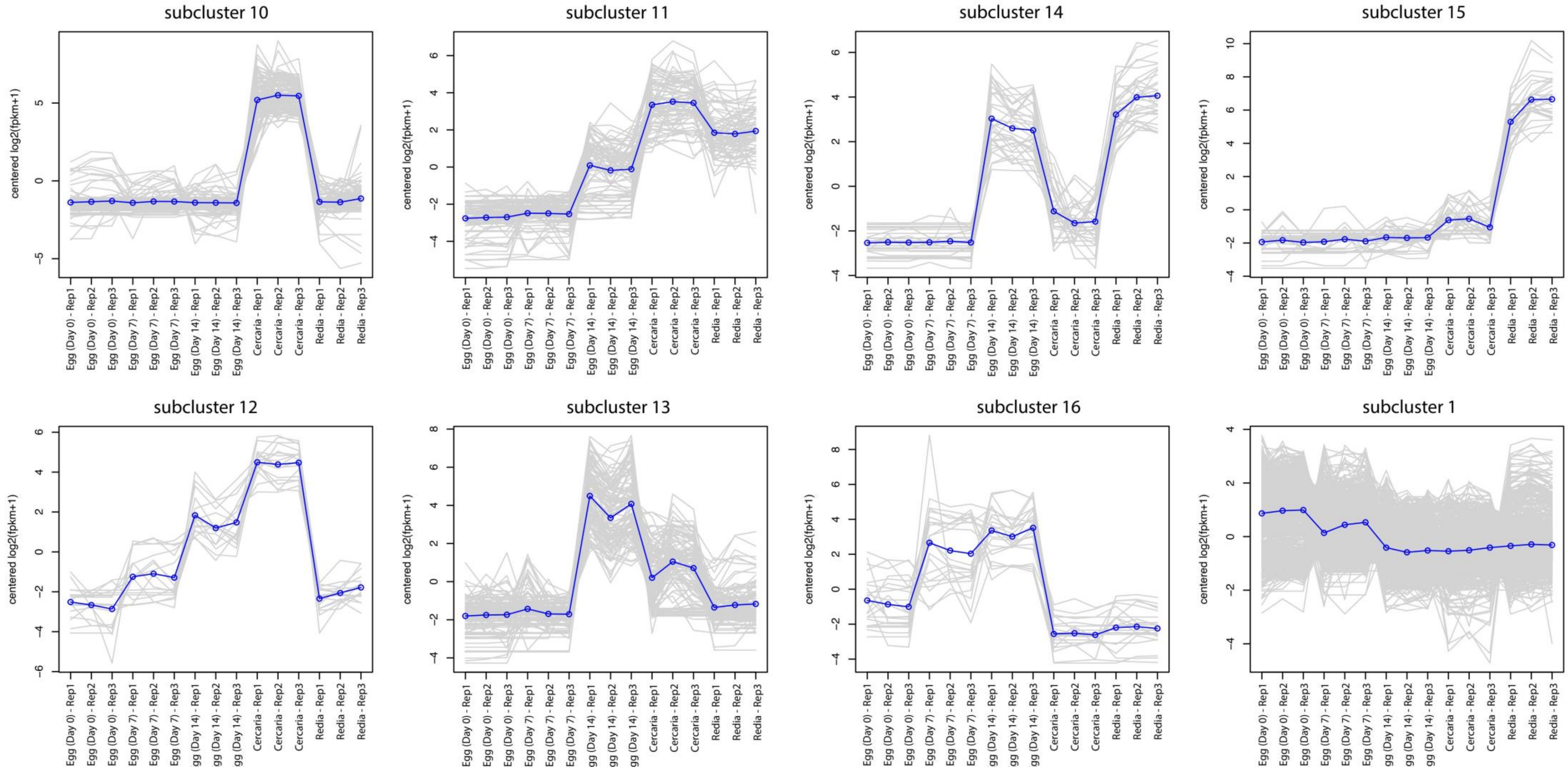

Supplementary Fig. S3: Expression profiles of all 16 subclusters. X-axis: replicates of the groups. Y-axis: log-transformed, median-centered FPKM values corresponding to changes in gene expression. Blue line represents the mean line, grey lines reflect individual genes.
